# Editorial: Molecular and genetic mechanisms of plant architecture regulation

**DOI:** 10.3389/fpls.2024.1421197

**Published:** 2024-05-13

**Authors:** Wenyi Wang, Weiwei Zhang, Muhammad Jamil, Jumin Tu, Lei Huang

**Affiliations:** ^1^ College of Agriculture, South China Agricultural University, Guangzhou, Guangdong, China; ^2^ Department of Botany and Plant Pathology, Purdue University, West Lafayette, IN, United States; ^3^ Department of Biotechnology and Genetic Engineering, Kohat University of Science and Technology, Kohat, Pakistan; ^4^ Institute of Crop Science, College of Agriculture and Biotechnology, Zhejiang University, Hangzhou, China; ^5^ Department of Economic Plants and Biotechnology, Yunnan Key Laboratory for Wild Plant Resources, Kunming Institute of Botany, Chinese Academy of Sciences, Kunming, China

**Keywords:** brassinosteroids, auxin, root system architecture, floral meristem, transcriptional factor

Plant architecture is an essential trait affecting crop growth, yield, and stress tolerance (Zhang et al., 2022). Ideotype breeding, which aims to modify the plant architecture, can accelerate crop breeding and ultimately increase the yield potential. The establishment of plant architecture is determined by complex interactions of regulatory networks involving miRNAs, phytohormones, key transcription factors (TFs), as well as metabolic and environmental factors (Guo et al., 2020). Accumulating studies on model plant species and crop plants have greatly improved our understanding of the physiological regulation of plant height, apical and axillary meristem activities, root and shoot branching, and the development of inflorescences and floral organs. Therefore, exploring the mechanisms underlying plant architecture regulation will deepen our understanding of fundamental plant science principles and facilitate crop breeding in modern agriculture. We have organized this Research Topic “Molecular and Genetic Mechanisms of Plant Architecture Regulation” in *Frontiers in Plant Science* to highlight discoveries in the molecular regulatory mechanisms underlying plant architecture. This Research Topic contains five original research articles and two reviews and has a broad scope across diverse plant species, including *Petunia*, mulberry (*Morus alba* L.), sugar beet (*Beta vulgaris* L.), rice (*Oryza sativa* L.), and *Arabidopsis.*

Brassinosteroids (BRs) are steroid hormones involved in various biological processes (Hussain et al., 2020). CONSTITUTIVE PHOTOMORPHOGENIC DWARF (CPD) is a pivotal enzyme in the BR biosynthesis pathway that plays an essential role in plant growth and development (Zhan et al., 2022). Guo et al. characterized and isolated *BvCPD* from *Beta vulgaris* L. *BvCPD* displayed strong expression in parenchymal cells and vascular bundles. Moreover, overexpression of *BvCPD* resulted in larger sugar beets compared to the wild type (WT), mainly due to the increased number and size of parenchyma cells and enlarged lumen and area of vessels in the xylem. Notably, *BvCPD* increased BR levels in the root, petiole, as well as leaf and regulated sugar beet root growth in cooperation with auxins and gibberellins. In plants, abscission is a highly programmed physiological process that is triggered by internal and external factors (Estornell et al., 2013). The *JOINTLESS* gene directly participates in developing a functional abscission zone (AZ) at the post-transcriptional level in tomato floral organs (Mao et al., 2000). Deng et al. identified MaABF1 (the ABA Binding Factor/ABA-Responsive Element Binding Proteins) and MaABI5 (Abscisic Acid-Insensitive 5) as the upstream regulators of the *MaJOINTLESS* gene in mulberry. MaABF1 transcriptionally activates *MaJOINTLESS*, while MaABI5 serves as a negative regulator, repressing the expression of *MaJOINTLESS* and several abscission-related genes, suggesting their potential distinct roles during mulberry fruit abscission.

Rice is a crucial cereal grain crop with a well-designed plant architecture, leading to high biomass production and grain yield (Wang and Li, 2005). Guo et al. revealed that *OseIF6.1* (*Eukaryotic Translation Initiation Factors 6.1*) encodes a eukaryotic translation initiation factor, regulating grain shape via cell expansion and proliferation. OseIF6.1, which interacts with OsNMD3 (Nonsense-Mediated mRNA Decay 3), is a conserved TF that encodes a nuclear export adaptor for the cytoplasmic maturation of pre-60S subunits. Notably, an *OsNMD3* knockdown mutant displayed phenotypic traits similar to those of *OseIF6.1*-RNAi plants; further research is necessary to understand their epistatic relationships during rice growth and development.

Mechanistic studies have also been performed on the model organism *Arabidopsis thaliana* in this topic. *Arabidopsis* hypocotyls are widely used to study the effects of hormones and plant growth factors on cell expansion, elongation, and division (Boron and Vissenberg, 2014). Favre et al. designed a mathematical model of auxin transport based on the movement of auxin by known polar and non-polar auxins, including ABCB1, ABCB19, and PINs, to investigate how shade avoidance syndrome is controlled by shallow auxin gradients along the hypocotyl. This impressive study simultaneously integrated experimental and mathematical modeling approaches to explore the mechanism whereby cellular growth patterns in *Arabidopsis* hypocotyls are controlled by auxin biosynthesis, transport, and catabolism. Drummond et al. explored the effects of strigolactone-independent mutants of *DAD2* in *Arabidopsis* and *Petunia* and found that varying the expression pattern of *DAD2* (*DECREASED APICAL DOMINANCE 2*) leads to the formation of distinct phenotypes from those of the WT and knockout mutants, suggesting that manipulation of the strigolactone receptor expression might be potentially useful for generating novel plant forms.

Indole-3-acetic acid (IAA) is involved in various aspects of root growth, including determining the direction of growth, promoting cell elongation, and facilitating the formation of lateral roots (Marchant et al., 2002). Jan et al. summarized the synergistical relationships between auxin and other phytohormones (mainly cytokinins and strigolactones) in different parts of the root system architecture, such as primary roots, lateral roots, and root hairs, suggesting that effects depend on specific hormone combinations and/or individual actions. This review also emphasizes the potential roles of phytohormone-regulated TFs during root development. In another review, Pelayo and Yamaguchi highlighted 3D gene expression atlases during floral meristem development. Additionally, the authors addressed the precise timing of floral meristem specifications and epigenetic regulation of determinacy. This review provides new insights into the well-established principles and discusses possible future directions for floral meristem development.

Overall, the compilation of articles included in this Research Topic provides novel insights into the molecular basis of plant architecture, with several articles highlighting the significance of phytohormones in plant growth and development. We believe that with an in-depth understanding of the molecular and genetic mechanisms underlying the regulation of plant architecture, it is possible to optimize crop yield.

## Author contributions

WW: Funding acquisition, Writing – original draft, Writing – review & editing. WZ: Writing – review & editing. MJ: Writing – review & editing. JT: Writing – review & editing. LH: Writing – original draft, Writing – review & editing, Funding acquisition.
